# Supplementary material for: Nasopharyngeal Carriage, Serotype Distribution, and Antimicrobial Susceptibility of Streptococcus pneumoniae Among PCV13-Vaccinated and -Unvaccinated Children in Iran
Source: Vaccines (Basel). 2025 Jun 29;13(7):707. doi: 10.3390/vaccines13070707 (PMC12299737; doi:10.3390/vaccines13070707)
Supplement: Supplementary file 1 [file vaccines-13-00707-s001.zip › vaccines-3668607-supplementary.pdf]

Table 1. Primer sequences used for confirming and serotyping of *S. pneumoniae* isolated from unvaccinated and PCV13 vaccinated children.

Primer		Sequence
lytA	Probe	FAM-TGCCGAAAACGCTTGATACAGGGAG-BHQ1
	forward	ACGCAATCTAGCAGATGAAGCA
	Reverse	TCGTGCGTTTTAATTCCAGCT
SP2020	Probe	FAM-AACCTTTGTTCTCTCTCGTGGCAGCTCAA-BHQ
	forward	TAAACAGTTTGCCTGTAGTCG
	Reverse	CCCGGATATCTCTTTCTGGA
cpsB	forward	GCA ATG CCA GAC AGT AAC CTC TAT
	Reverse	CCT GCC TGC AAG TCT TGA TT

Table 2. Comparing socio-demographic characteristics between *S. pneumoniae* carrier and non-carrier children.

**Variables**		**Unvaccinated N (%)**			**Vaccinated N (%)**		
		**Non-carriage**	**carriage**	**P**	**Non-carriage**	**carriage**	**P**
**Area (Tehran)**	no	56 (84.8)	10 (15.2)	0.061	8 (88.9)	1 (11.1)	0.437
	yes	19 (67.9)	9 (32.1)		70 (77.8)	20 (22.2)	
**Sharing bedroom with more than 2 persons**	no	20 (76.9)	6 (23.1)	0.828	43 (79.6)	11 (20.4)	0.954
	yes	60 (78.9)	16 (21.1)		38 (79.2)	10 (20.8)	
**Sharing bedroom with parents**	no	22 (73.3)	8 (26.7)	0.419	43 (76.8)	13 (23.2)	0.469
	yes	58 (80.6)	14 (19.4)		38 (82.6)	8 (17.4)	
**Exposure to cigarette smoke**	no	47 (74.6)	16 (25.4)	0.232	70 (83.3)	14 (16.7)	0.34
	yes	33 (84.6)	6 (15.4)		11 (61.1)	7 (38.9)	

**presence of person 60**	no	70 (79.5)	18 (20.5)	0.495+	76 (78.4)	21 (21.6)	0.581+
	yes	10 (71.4)	4 (28.6)		5 (100.0)	0 (0.0)	
**Presence of older sibling**	no	5 (55.6)	4 (44.4)	0.138	11 (84.6)	2 (15.4)	0.276+
	yes	34 (79.1)	9 (20.9)		17 (65.4)	9 (34.6)	
**Presence of both older and younger sibling**	no	34 (72.3)	13 (27.7)	0.314+	26 (70.3)	11 (29.7)	1.000+
	yes	5 (100.0)	0 (0.0)		2 (100.0)	0 (0.0)	
**Number of sibling**	0	29 (85.3)	5 (14.7)	0.486+	51 (83.6)	10 (16.4)	0.221+
	1-2	46 (75.4)	15 (24.6)		30 (73.2)	11 (26.8)	
	>=3	5 (83.3)	1 (16.7)		0 (0.0)	0 (0.0)	
**Previous hospitalization**	no	58 (81.7)	13 (18.3)	0.226	40 (76.9)	12 (23.1)	0.526
	yes	22 (71.0)	9 (29.0)		41 (82.0)	9 (18.0)	
**Cause of hospitalization**	non-infectious	16 (80.0)	4 (20.0)	1.000+	22 (78.6)	6 (21.4)	0.716+
	infectious	5 (83.3)	1 (16.7)		17 (85.0)	3 (15.0)	
**Antibiotic treatment within the last 3 months**	no	74 (81.3)	17 (18.7)	**0.041**	49 (84.5)	9 (15.5)	0.216+
	yes	6 (54.5)	5 (45.5)		32 (72.7)	12 (27.3)	
**Previous respiratory infections**	no	79 (81.4)	18 (18.6)	**0.007+**	69 (81.2)	16 (18.8)	0.335+
	yes	1 (20.0)	4 (80.0)		12 (70.6)	5 (29.4)	

+Fisher's Exact Test. Other: Pearson Chi square test. Bold P values are shown as significant.

Table 3. Comparison of serotype distribution based on the number of resistance classes among *S. pneumoniae* isolated from PCV13 vaccinated and unvaccinated children

**Serotype/Serogroup**	**Unvaccinated**				**Vaccinated**			
	**Number of resistant Classes**				**Number of resistant Classes**			
	**0**	**1**	**2**	**MDR**	**0**	**1**	**2**	**MDR**
	**N (%)**	**N (%)**	**N (%)**	**N (%)**	**N (%)**	**N (%)**	**N (%)**	**N (%)**
**19F**	1 (25.0)	1 (25.0)	1 (9.1)	0 (0.0)	0 (0.0)	1 (33.3)	2 (18.2)	1 (100.0)
**15B/15C**	0 (0.0)	0 (0.0)	1 (9.1)	0 (0.0)	0 (0.0)	1 (33.3)	4 (36.4)	0 (0.0)
**23F**	0 (0.0)	0 (0.0)	2 (18.2)	1 (33.3)	0 (0.0)	0 (0.0)	0 (0.0)	0 (0.0)
**35F/47F**	0 (0.0)	0 (0.0)	0 (0.0)	0 (0.0)	1 (25.0)	1 (33.3)	1 (9.1)	0 (0.0)

**9V/9A**	1 (25.0)	0 (0.0)	1 (9.1)	0 (0.0)	0 (0.0)	0 (0.0)	1 (9.1)	0 (0.0)
**14**	1 (25.0)	0 (0.0)	1 (9.1)	0 (0.0)	0 (0.0)	0 (0.0)	0 (0.0)	0 (0.0)
**23A**	0 (0.0)	0 (0.0)	1 (9.1)	0 (0.0)	0 (0.0)	0 (0.0)	1 (9.1)	0 (0.0)
**6E/6B**	0 (0.0)	0 (0.0)	1 (9.1)	1 (33.3)	0 (0.0)	0 (0.0)	0 (0.0)	0 (0.0)
**11A/11D/18F**	0 (0.0)	0 (0.0)	1 (9.1)	0 (0.0)	0 (0.0)	0 (0.0)	0 (0.0)	0 (0.0)
**17F/33C**	0 (0.0)	0 (0.0)	0 (0.0)	0 (0.0)	1 (25.0)	0 (0.0)	0 (0.0)	0 (0.0)
**18B/18C**	0 (0.0)	1 (25.0)	0 (0.0)	0 (0.0)	0 (0.0)	0 (0.0)	0 (0.0)	0 (0.0)
**19A**	0 (0.0)	0 (0.0)	1 (9.1)	0 (0.0)	0 (0.0)	0 (0.0)	0 (0.0)	0 (0.0)
**22F/22A**	0 (0.0)	0 (0.0)	0 (0.0)	0 (0.0)	1 (25.0)	0 (0.0)	0 (0.0)	0 (0.0)
**3**	0 (0.0)	0 (0.0)	0 (0.0)	0 (0.0)	1 (25.0)	0 (0.0)	0 (0.0)	0 (0.0)
**4**	0 (0.0)	1 (25.0)	0 (0.0)	0 (0.0)	0 (0.0)	0 (0.0)	0 (0.0)	0 (0.0)
**6A**	0 (0.0)	1 (25.0)	0 (0.0)	0 (0.0)	0 (0.0)	0 (0.0)	0 (0.0)	0 (0.0)
**6B**	0 (0.0)	0 (0.0)	1 (9.1)	0 (0.0)	0 (0.0)	0 (0.0)	0 (0.0)	0 (0.0)
**6C/6B**	1 (25.0)	0 (0.0)	0 (0.0)	0 (0.0)	0 (0.0)	0 (0.0)	0 (0.0)	0 (0.0)
**9N/9L**	0 (0.0)	0 (0.0)	0 (0.0)	0 (0.0)	0 (0.0)	0 (0.0)	1 (9.1)	0 (0.0)
**9V**	0 (0.0)	0 (0.0)	0 (0.0)	0 (0.0)	0 (0.0)	0 (0.0)	1 (9.1)	0 (0.0)
**NT**	0 (0.0)	0 (0.0)	0 (0.0)	1 (33.3)	0 (0.0)	0 (0.0)	0 (0.0)	0 (0.0)
